# Understanding On-Campus Interactions With a Semiautomated, Barcode-Based Platform to Augment COVID-19 Contact Tracing: App Development and Usage

**DOI:** 10.2196/24275

**Published:** 2021-03-26

**Authors:** Thomas Foster Scherr, Austin N Hardcastle, Carson Paige Moore, Jenna Maria DeSousa, David Wilson Wright

**Affiliations:** 1 Department of Chemistry Vanderbilt University Nashville, TN United States

**Keywords:** contact tracing, COVID-19, disease surveillance, digital health

## Abstract

**Background:**

The COVID-19 pandemic has forced drastic changes to daily life, from the implementation of stay-at-home orders to mandating facial coverings and limiting in-person gatherings. While the relaxation of these control measures has varied geographically, it is widely agreed that contact tracing efforts will play a major role in the successful reopening of businesses and schools. As the volume of positive cases has increased in the United States, it has become clear that there is room for digital health interventions to assist in contact tracing.

**Objective:**

The goal of this study was to evaluate the use of a mobile-friendly app designed to supplement manual COVID-19 contact tracing efforts on a university campus. Here, we present the results of a development and validation study centered around the use of the MyCOVIDKey app on the Vanderbilt University campus during the summer of 2020.

**Methods:**

We performed a 6-week pilot study in the Stevenson Center Science and Engineering Complex on Vanderbilt University’s campus in Nashville, TN. Graduate students, postdoctoral fellows, faculty, and staff >18 years who worked in Stevenson Center and had access to a mobile phone were eligible to register for a MyCOVIDKey account. All users were encouraged to complete regular self-assessments of COVID-19 risk and to key in to sites by scanning a location-specific barcode.

**Results:**

Between June 17, 2020, and July 29, 2020, 45 unique participants created MyCOVIDKey accounts. These users performed 227 self-assessments and 1410 key-ins. Self-assessments were performed by 89% (n=40) of users, 71% (n=32) of users keyed in, and 48 unique locations (of 71 possible locations) were visited. Overall, 89% (202/227) of assessments were determined to be low risk (ie, asymptomatic with no known exposures), and these assessments yielded a CLEAR status. The remaining self-assessments received a status of NOT CLEAR, indicating either risk of exposure or symptoms suggestive of COVID-19 (7.5% [n=17] and 3.5% [n=8] of self-assessments indicated moderate and high risk, respectively). These 25 instances came from 8 unique users, and in 19 of these instances, the at-risk user keyed in to a location on campus.

**Conclusions:**

Digital contact tracing tools may be useful in assisting organizations to identify persons at risk of COVID-19 through contact tracing, or in locating places that may need to be cleaned or disinfected after being visited by an index case. Incentives to continue the use of such tools can improve uptake, and their continued usage increases utility to both organizational and public health efforts. Parameters of digital tools, including MyCOVIDKey, should ideally be optimized to supplement existing contact tracing efforts. These tools represent a critical addition to manual contact tracing efforts during reopening and sustained regular activity.

## Introduction

SARS-CoV-2, the virus that causes COVID-19, first emerged in late 2019. Months into the pandemic, the spread of COVID-19 continues to affect the world at large [1,2]. In response to COVID-19, entire countries enacted sweeping measures both nationally and in local hot spots. While these actions varied from country to country; in the United States, the declaration of a public health emergency led many state and local governments to implement “stay-at-home” directives, among other guidelines [3-6]. The ramifications were felt on state, city, and community levels; the consequences of these decisions included the closing of many nonessential businesses and a shift to remote work for many employees. Similarly, universities across the country closed research laboratories, removed undergraduate students from campus, and transitioned to virtual classrooms.

**Figure 1:**
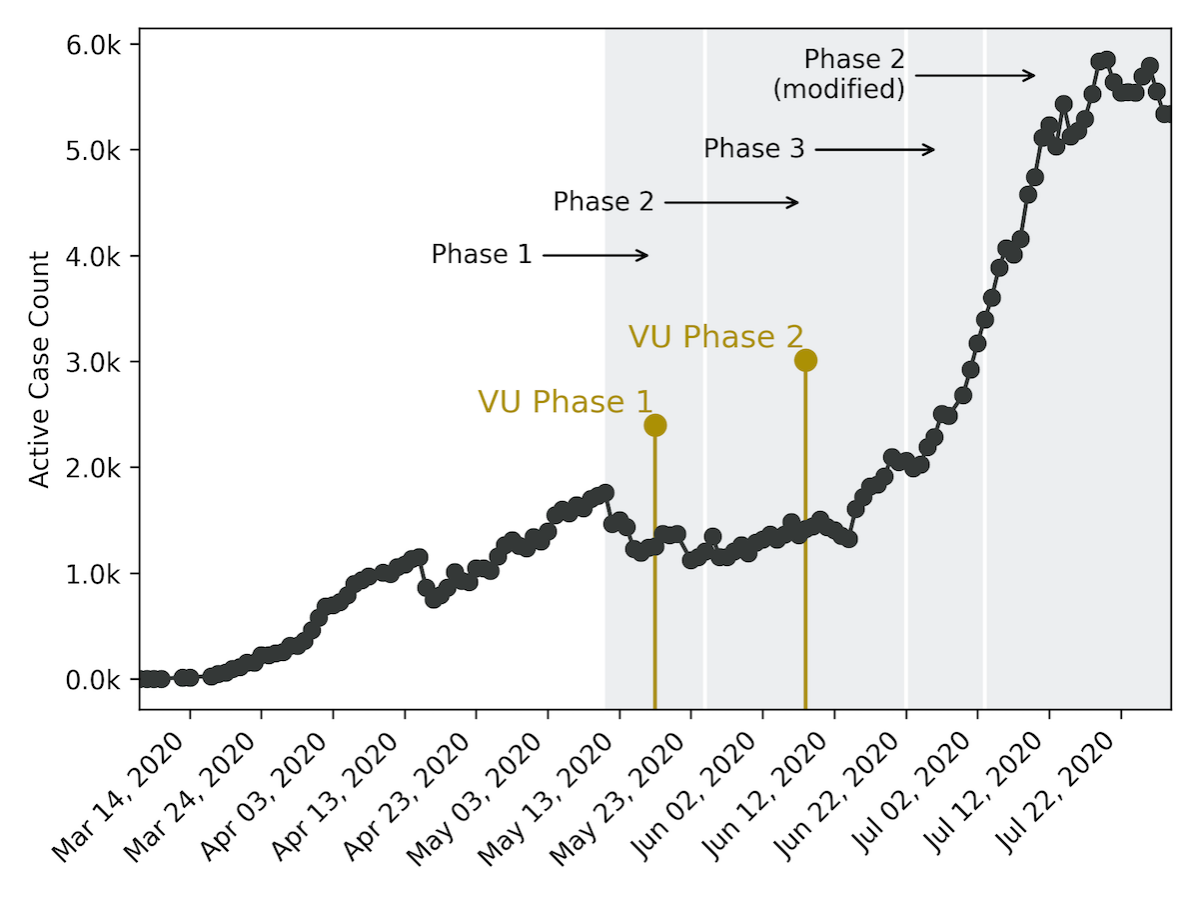
Active COVID-19 cases in Davidson County, TN, from mid-March through July 2020. Gray shaded boxes indicate the phases of the Nashville Metro Government reopening plan, while the gold lines indicate the start date of each phase of Vanderbilt University’s (VU) reopening plan.

In Nashville, TN, the local government laid out a phased reopening of the city after the end of a stay-at-home order, which extended beyond the restrictions at the state level [7]. Phase 1, which began on May 11, allowed retail stores, restaurants, and bars serving food to open at 50% capacity, while high-touch and high-contact businesses such as nail salons, gyms, and entertainment venues remained closed. In phase 1, the Nashville Metro government encouraged social distancing and recommended, but did not require, face masks. Nashville’s phase 2 of reopening began on May 25, increasing restaurant and retail capacity to 75% and opening high-touch businesses and entertainment venues at 50% and limited capacity, respectively. On June 22, Nashville entered phase 3 of the Metro reopening plan, although the city rolled back into a modified phase 2 stage on July 3 after a spike in cases (Figure 1) [8].

At Vanderbilt University, similar phased reopening steps were taken [9]. Each phase on campus mandated social distancing and masks, utilized on-campus pedestrian traffic plans, and encouraged remote work from staff or students when possible. The university entered phase 1 of their reopening on May 18, allowing research activities to resume at 33% capacity. On June 8, the university entered phase 2, allowing research capacity to increase from 33% to 50%, provided that 6 feet of social distance could be maintained between workers or students.

As states across the country begin to relax their precautionary measures and resume educational activities in the fall, it is generally understood that there is a need for increased vigilance and precautionary steps [10-12]. Many organizations are utilizing symptom-tracking software to monitor their community members during the reopening process, including in workplaces and on college campuses. Many freely available risk assessments have been widely distributed by public health entities, for-profit technology companies, and for-profit health care systems. While these are useful as informational tools and for understanding health disparities, there are concerns over the accuracy and utility of self-report symptom trackers in reopening efforts given the high degree of asymptomatic transmission associated with the current pandemic [13-16]. This highlights the need for other tools to focus on how to limit the spread from unknown transmission events.

Contact tracing has been a necessary method of identifying potential exposure events and understanding the epidemiology of the novel virus [17-24]. However, months into the pandemic, contact tracing remains largely a manual and labor-intensive process in which health care workers interview confirmed-positive COVID-19 incident cases and gather information on exposed people and locations. As case volumes grow and manual efforts struggle to handle the increase, it is clear that digital technology could assist with this process [25-28]. For instance, Apple and Google have partnered on a passive system that utilizes Bluetooth signals on mobile devices to identify when users are within a given distance for a certain time (a “contact event”) [27]. While Apple and Google have implemented best-in-class enhanced security features (eg, decentralized storage, rotating keys), security vulnerabilities have been identified in other strategies that rely exclusively on Bluetooth signals without similar protections in place [29-32]. Others have developed similar systems that utilize continuous GPS monitoring [25]. These approaches have raised substantial data ownership and privacy concerns, and early reports suggest that Bluetooth and GPS may struggle to accurately identify true contact through walls or on different floors of the interior floorplans common to office buildings and college campuses [33-38].

In response to these concerns, we developed MyCOVIDKey as an alternative digital contact tracing tool based on a combination of recurring risk assessments and a location check-in strategy. Since it relies on discrete event monitoring rather than continuous location monitoring or potentially vulnerable Bluetooth broadcasts, this approach is an alternative to current strategies and can provide an automated solution to supplement manual contact tracing efforts. The key-in feature of MyCOVIDKey, where users scan a location-specific barcode, can, importantly, augment existing contact tracing efforts in the face of asymptomatic transmission or inaccurate and unreliable symptom assessments. In this paper, we describe an app viability study in which we sought to understand the usefulness of this platform, its potential efficacy, and the sensitivity of its parameters.

## Methods

### Study Design

The Stevenson Center Science and Engineering Complex (Stevenson Center) of Vanderbilt University’s campus in Nashville, TN, was chosen as the study setting. Stevenson Center consists of 8 buildings in close proximity to one another. The buildings contain classrooms, research and teaching laboratories, graduate student and faculty offices, an engineering library (closed for the duration of the pilot study), and departmental administration offices. The buildings all have multiple floors, dedicated entrances and exits, stairwells, and elevators; several of the buildings are interconnected. For these reasons, Stevenson Center makes an ideal proxy for campuses at large, as well as moderately sized office complexes.

**Figure 2:**
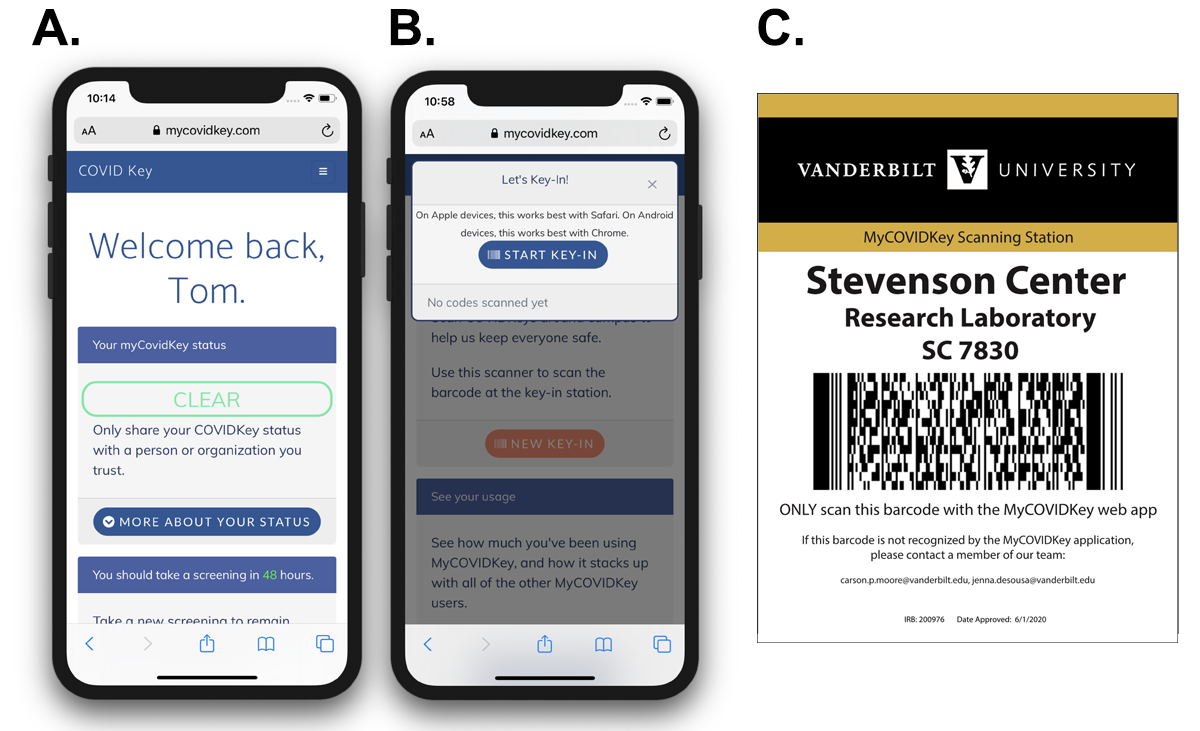
(A) The landing page of MyCOVIDKey, shown after a successful login. (B) A pop-up modal window that enables users to key in by scanning a location’s bar code flyer. (C) A representative key-in flyer, with a barcode that has a unique embedded hash code specific to a location on campus.

**Figure 3:**
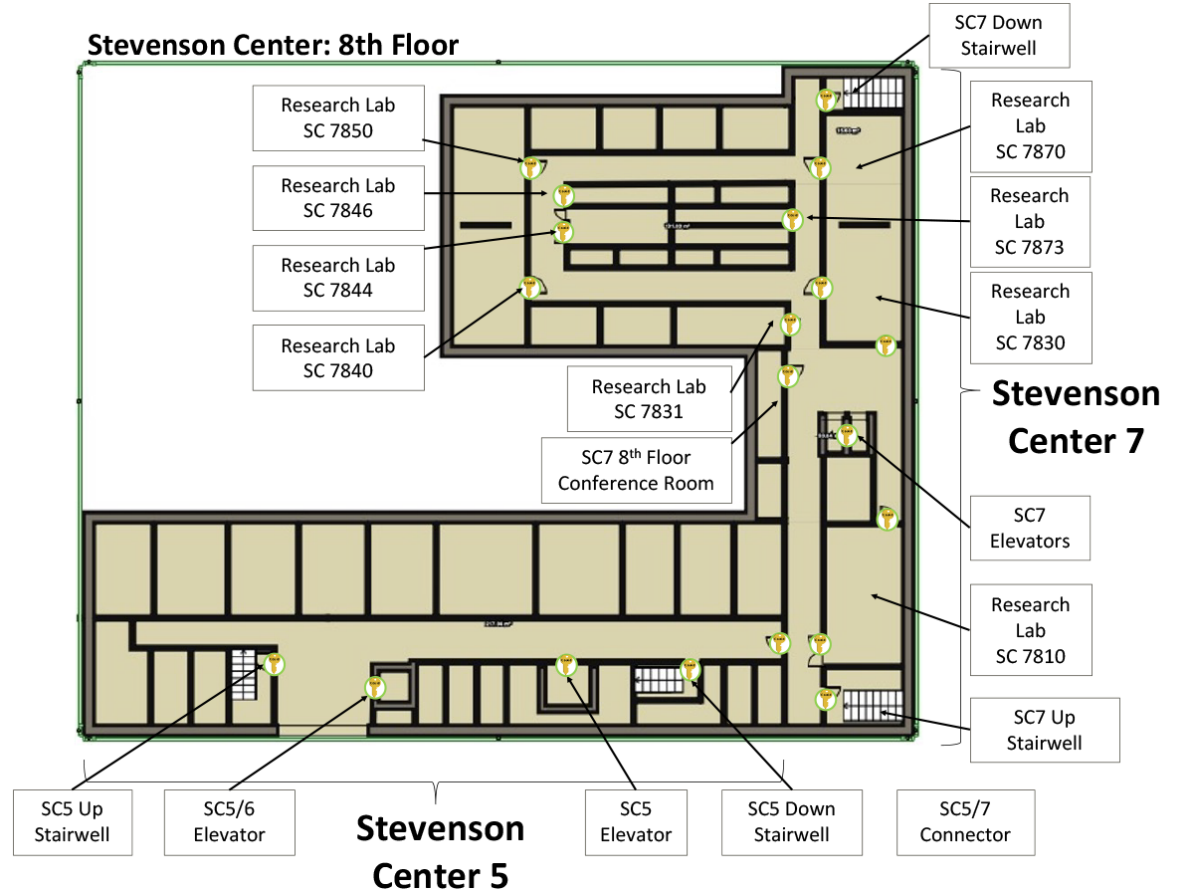
A coverage map of key-in flyers on the 8th floor of Stevenson Center (SC) 5 and Stevenson Center 7.

Laminated flyers (Figure 2C) were fixed to walls near building, stairwell, and elevator entrances, as well as in most common rooms and laboratories where users were expected to have returned to once on campus (Figure 3). Each flyer contained a barcode with a data payload of a unique hash code specific to that particular location. We elected to use PDF417 barcodes, commonly used on identification cards, instead of more common barcode types (ie, QR [quick response] code, data matrix). We believed that selecting a less common barcode that is not typically used to encode web addresses would have a positive impact on security by avoiding barcode hijacking (where a barcode is covered by another barcode that redirects a user to a malicious website) and requiring users to use our app instead of their mobile devices’ native camera app (most of which do not natively decode PDF417 barcodes). In total, there were 71 coded locations throughout the different buildings.

The study was set for 6 weeks and began on June 17, 2020. Participants were recruited via flyers posted throughout Stevenson Center as well as department-wide email lists. Users were provided brief instructions via a guided walk-through of the app the first 4 times that they arrived at the home screen. A weekly raffle based on usage was put in place as an incentive; however, all users were free to use the app at will. Upon completion of the pilot study, a survey was sent to all participants. This survey included questions about user demographics, as well as satisfaction questions focused on the MyCOVIDKey user experience. This work focuses on the technical implementation and results from the pilot; a thorough analysis of the postpilot survey, as well as a usability analysis and recommendations for improvement, are described elsewhere [39].

This study was reviewed and approved by the Vanderbilt University Institutional Review Board (#200976) on June 1, 2020.

### App Design and Use

The MyCOVIDKey app was hosted by Amazon Web Services [40]. The platform consists of an Apache HTTP web server, a MySQL database, a custom-built PHP application programming interface, and a responsive, mobile-friendly (JavaScript, CSS, HTML) frontend. All data transmission between the server and client devices used secure protocols (HTTPS/SSL). A custom-built paradata capture library was included to perform usage analytics.

The app has a user hierarchy that includes specific privileges for four different classes of users: users, app administrators, contact tracers, and developers (Multimedia Appendix 1). All created accounts are users by default, with additional privileges accessible only if they have been granted by someone with the higher privilege level. With this structure, app administrator and contact tracer are distinct roles: the former sets parameters for use within the app but does not access user data; the latter performs the actual contact tracing with access to identifying information. This user hierarchy builds a foundation for enhanced privacy features where identifying user data can be siloed from deidentified but linked key-in and symptom information. Such an approach, which will be integrated prior to a wider rollout, would follow the lead set by the Apple/Google platform by saving different pieces of data on isolated servers that are managed by distinct user classes. Only in the event of a positive test will the user hierarchy coordinate to access the data necessary for contact tracing.

**Figure 4:**
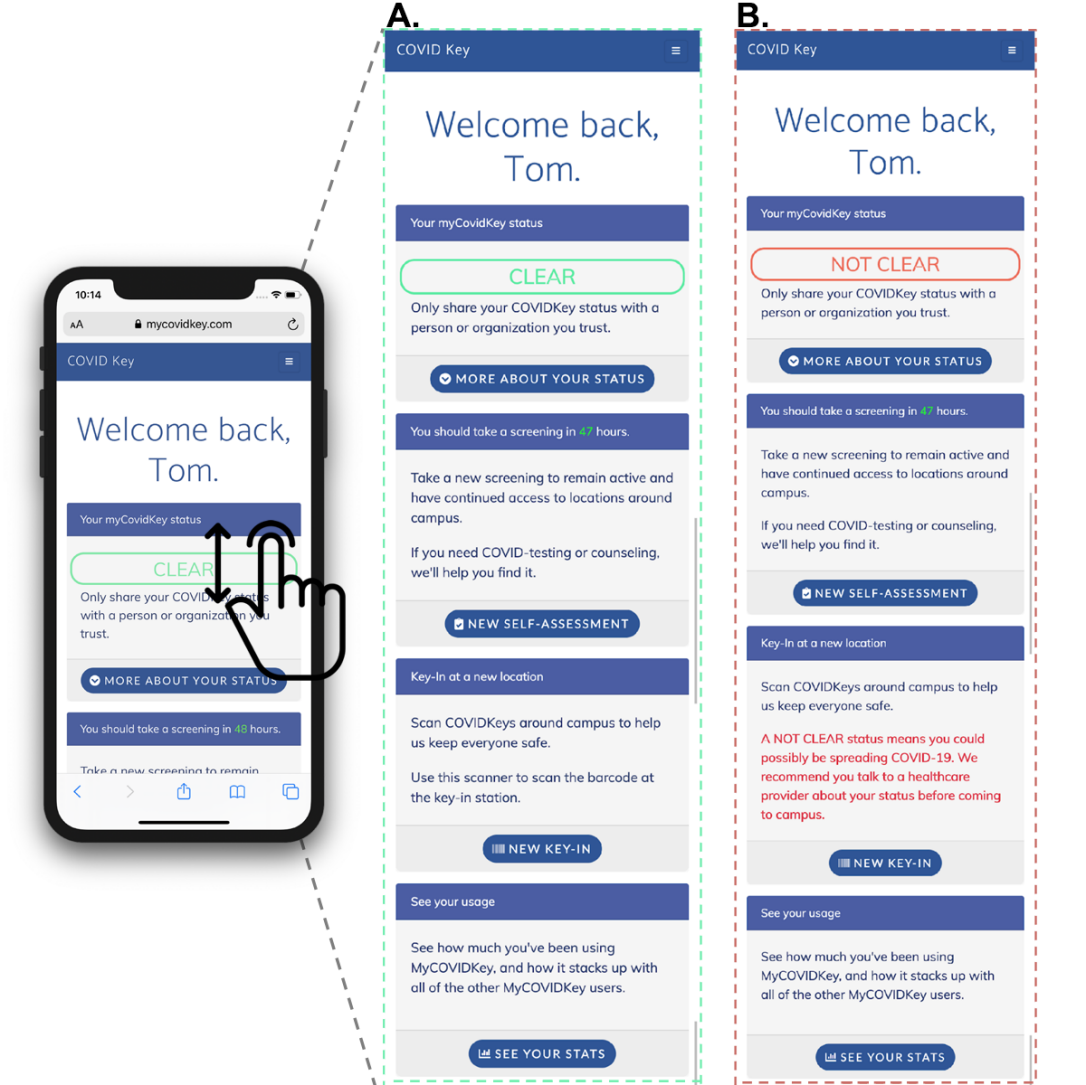
The home screen of MyCOVIDKey displays information about the user’s current MyCOVIDKey status, allows users to perform self-assessments, key in to new locations, and view simple usage statistics. Certain features are disabled, and the text is adjusted to reflect a user’s current status: (A) CLEAR, (B) NOT CLEAR.

**Figure 5:**
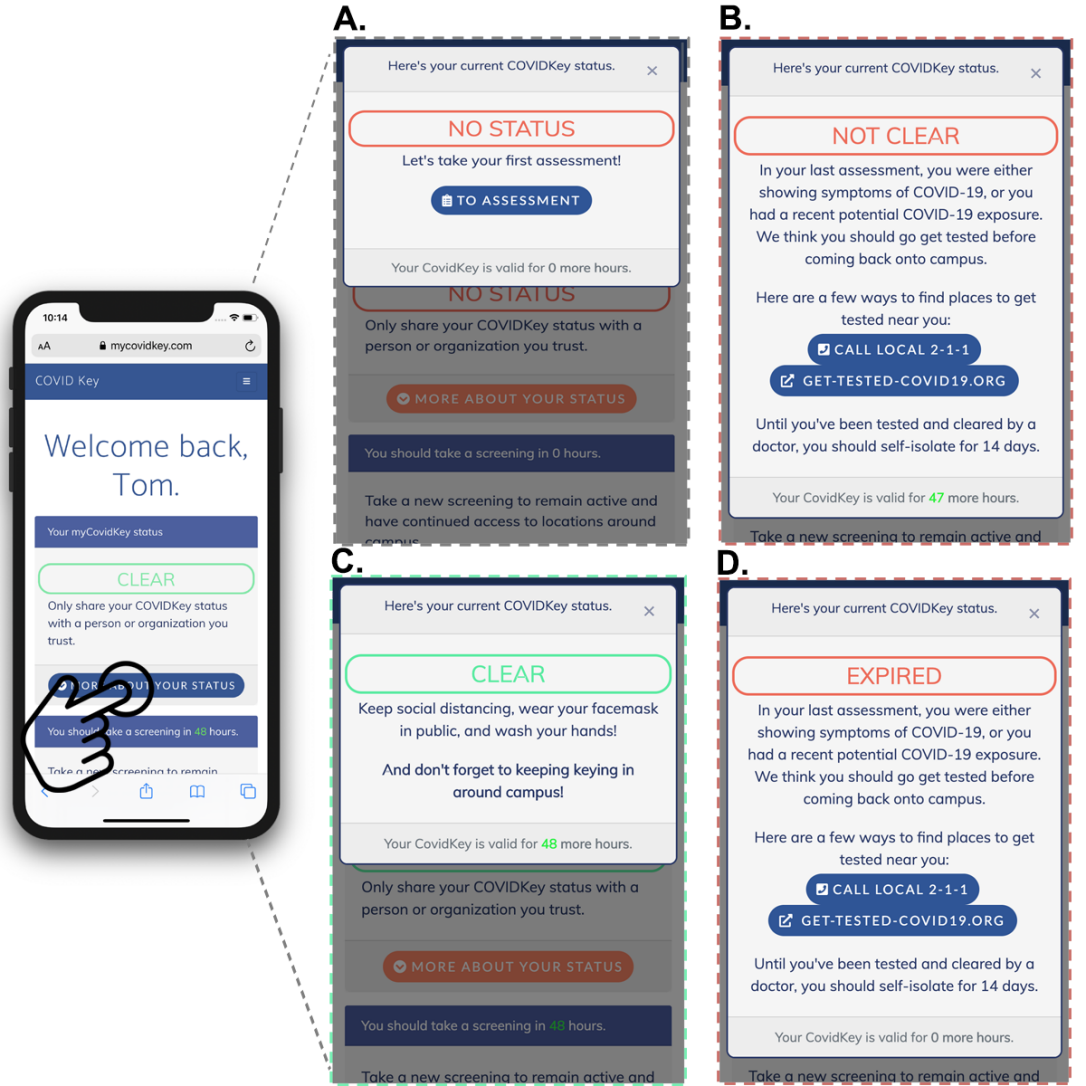
Recommendations were customized based on the user’s current status: (A) NO STATUS, (B) NOT CLEAR, (C) CLEAR, and (D) EXPIRED.

During account creation, participants provided an email address, password, phone number, name, birth date, and home zip code. Demographic data (age, sex, race) were not collected from users upon creation of a MyCOVIDKey account. After a successful login, users were directed to the landing page (Figures 2A and 4, and Multimedia Appendix 2). On this screen, separate tiles (Figure 5) could be expanded to display information on the user’s current MyCOVIDKey status (including recommendations based on their most recent self-assessment), start a new self-assessment, present a modal window to perform barcode scanning at MyCOVIDKey locations, compare an individual’s usage statistics to the entire cohort, and display their progress for the weekly raffle.

**Figure 6:**
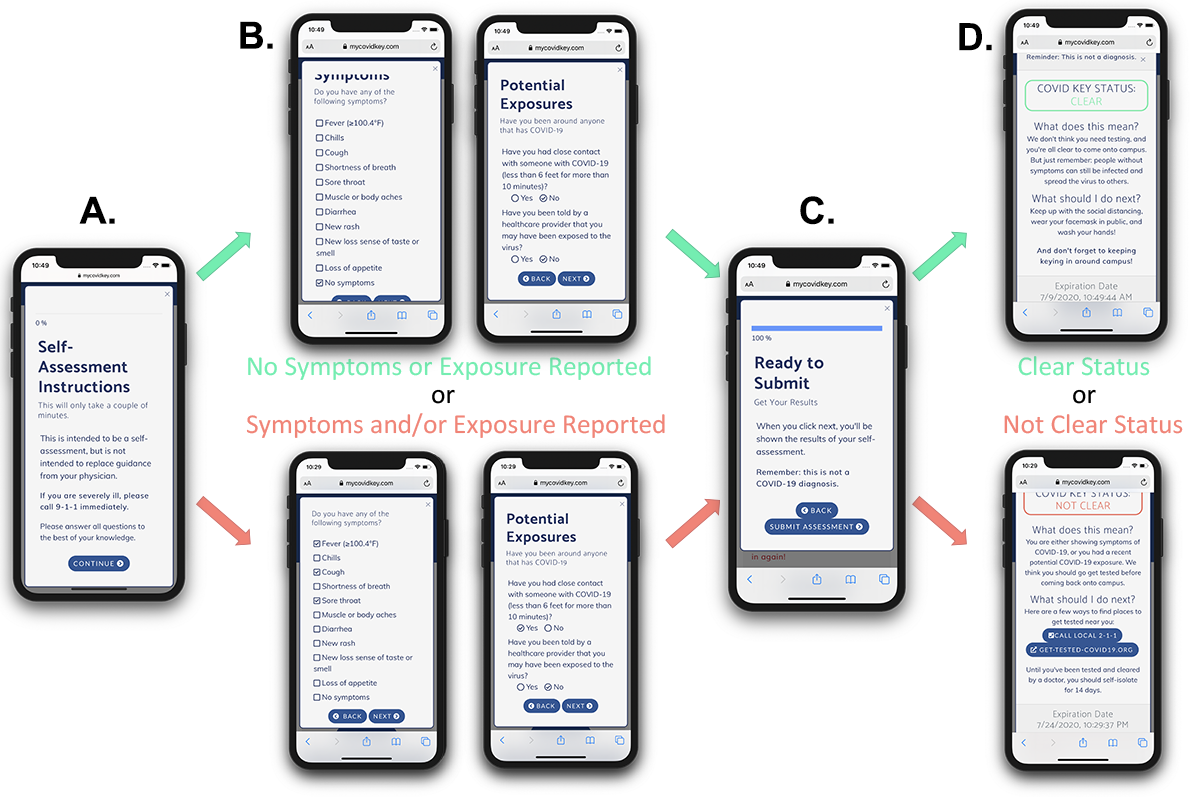
The modal window to perform a self-assessment shows (A) brief instructions, (B) common symptoms and exposure risks of COVID-19, (C) a confirmation/submission screen, and (D) customized results based on the outcome of the self-assessment. Potential pathways to CLEAR and NOT CLEAR statuses are shown on top (green) and bottom (red), respectively.

The self-assessment was designed to be brief, since it was intended to be used repetitively, yet included COVID-19 symptoms outlined by the Centers for Disease Prevention and Control (CDC), as well as two questions designed to determine exposure risk. Symptom- and exposure-free users were given a status of CLEAR while a selection of any symptom or exposure would designate a status as NOT CLEAR (Figure 6). Although the user-facing result of the self-assessment was binary, internally self-assessments were coded using a point-based system to classify results as “low,” “moderate,” or “high.” Our scoring system counted canonical COVID-19 symptoms (fever, chills, cough, and shortness of breath) and known exposure risks as 3 points; the presence of a rash or loss of smell and/or taste counted as 2 points; and a sore throat, body aches, and diarrhea were scored as 1 point. After summing the individual point values, the risk score was classified as follows: 0 points was defined as low risk, greater than 0 but less than 3 was defined as moderate risk, and greater than or equal to 3 was defined as high risk. While there are many ongoing efforts to distill qualitative COVID-19 symptoms to a numerical risk score, there currently is no standard approach for doing so. As such, the scoring system that we adopted proved useful to numerically differentiate users with canonical symptoms of COVID-19 from those with less specific symptoms.

Users with a CLEAR status were provided social support and encouragement to stay vigilant; those that received a NOT CLEAR status were instructed that the self-assessment was not a diagnosis and that they should seek diagnostic testing prior to returning to campus. The latter group was provided with a link to locate testing resources based on the zip code that they provided when their account was created [41]. When a self-assessment was completed, the user ID, symptoms, potential exposures, and the time stamp of the self-assessment were recorded. For this study, assessments were given an expiration of 48 hours, after which the key-in feature of the app was disabled until the user took a new self-assessment. This duration was chosen to increase the likelihood of continued usage by minimizing the burden on users during the pilot. However, the frequency of recurring self-assessments could readily be customized by organizational administrators to meet their needs. Upon completing a new self-assessment, the key-in feature was reactivated.

When a user entered a location with a key-in flyer, they could click the “New Key-In” button on the home screen to launch the key-in modal window. From there, the user was prompted to press the “Start Key-In” button, which initiated the barcode scanner (using the Scandit Software Development Kit, v5.0-5.1). When a user scanned a barcode, the app collected that event in the database, recording the user ID of the scanner, the time stamp of the scan, and the location ID that was scanned.

A weekly raffle was implemented on June 23 to incentivize participation. Users were allowed to accumulate entries in the drawing based on the number of self-assessments they performed and their number of key-ins each week. The number of entries was weighted for each event: each self-assessment was worth 10 entries in the raffle, and each key-in was worth 1 entry in the raffle. To avoid attempts to manipulate raffle outcomes by increased usage, the maximum number of entries a user could receive for each type of event was limited to 30.

Administrator features were included that allowed the study team to visualize usage metrics on a dashboard, perform manual contact tracing queries, and see results from the automated contact tracing algorithm. This algorithm is visually depicted in Multimedia Appendix 3. Briefly, when a participant completes a self-assessment that indicates either symptoms of or potential exposure to COVID-19, that creates a “person of interest” (POI) case. A case window is created that extends 48 hours prior to the causative self-assessment time stamp (the reverse case window) and continues for 14 days after the self-assessment (the forward case window). Any locations that the user keys in to during this period become “locations of interest.” A second window of ±30 minutes is then created, centered around the time stamp of the POI’s key-in at a particular location (the “contact overlap window”). Any other users who key in to the same location during the overlap window are deemed “contacts of interest.” It is important to emphasize that these criteria are not the same as the CDC’s guidelines for “close contact”; instead, our approach aligns with the goal of streamlining manual contact tracing efforts, rather than replacing them. As such, the lengths of the forward case window, the reverse case window, and the contact overlap window can be customized based on organizational rules, manual contact tracing infrastructure and bandwidth, and location type.

### Data Analysis

The data that were collected consisted of user information, the results of recurring self-assessments, data from key-ins, as well as app (usage) paradata. At the conclusion of the 6-week pilot, the data were exported from the database for analysis using Python statistical and visualization packages (Python Software Foundation). The data were then coded, identifiers removed, and then loaded into a REDCap project for long-term storage.

## Results

### Overall Usage

Over the 6-week pilot period, 45 participants created accounts. While our participants were not entirely from a single department, the majority were affiliated with the Department of Chemistry. For context, the Department of Chemistry has approximately 210 graduate students, postdoctoral fellows, faculty, and staff. During Phase 1 of the reopening, while operating at 33% capacity, 69 people were allowed to occupy space within the department; while at 50% capacity, this number increased to 105 people.

**Figure 7:**
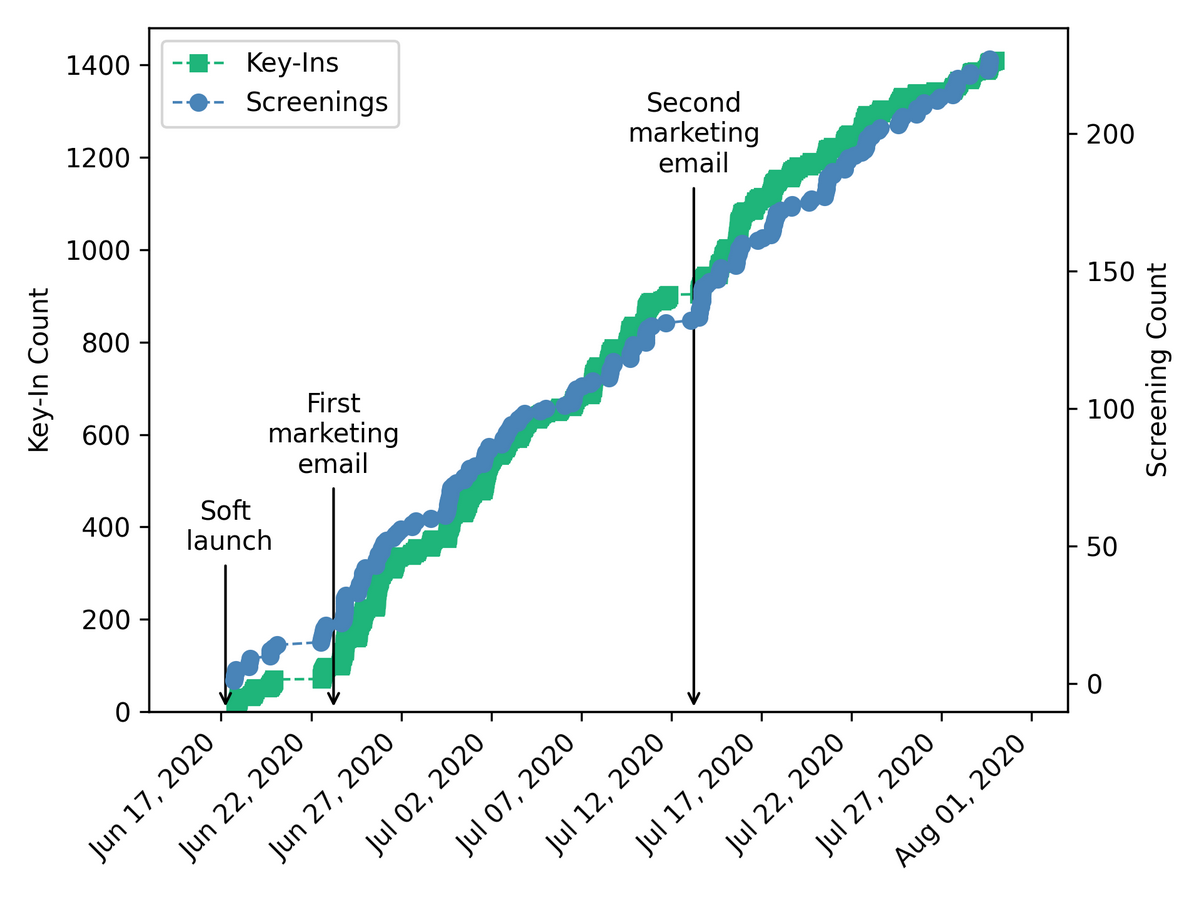
Usage of key-ins and screenings throughout the duration of the study along with key project events.

Of the 45 created accounts, 43 users logged in to the app at least once. These participants performed a total of 227 self-assessments and keyed in 1410 times at 48 distinct locations. Our soft launch period resulted in modest participant enrollments and app usage (Figure 7). On June 23, the first recruitment email was sent and the weekly raffle was instituted, and both participant sign-ups and app usage increased substantially. A second recruitment email was sent out approximately mid-way through the study (timed to avoid conflict with the July 4 holiday closure); however, it had little impact on app usage.

In the following sections, we analyze the self-assessments, key-ins, and contact tracing cases that resulted from this usage. Of the 45 individual users, only 26 completed the follow-up survey in its entirety, and 4 returned the survey incomplete (n=30, 66.6%). A total of 15 users did not complete the final follow-up survey. All of the users who completed the survey in some capacity provided demographic information including age, race, and gender.

### Self-assessment and Key-In Usage

**Figure 8:**
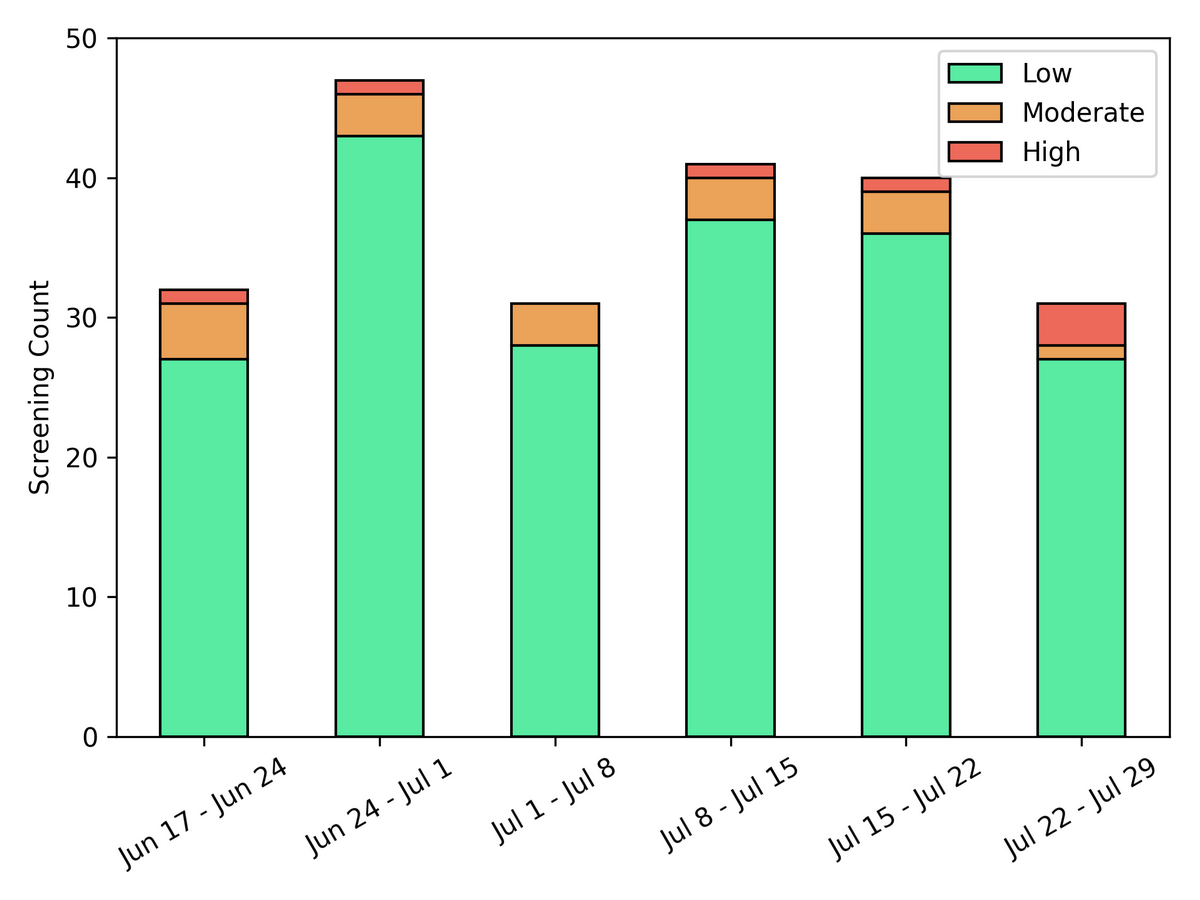
Weekly counts of user self-assessments classified as low, moderate, or high risk.

Self-assessments were performed by 89% (40/45) of users. The majority of the assessments (202/227, 89%) indicated low risk (ie, asymptomatic with no known exposures), 7.5% (17/227) of self-assessments were of moderate risk (ie, nonzero scores less than 3), and 3.5% (8/227) of self-assessments were of high risk (ie, scores of 3 or more) (Figure 8). Accounting for the different dates of user account creation, users performed 1.02 self-assessments per week (Multimedia Appendix 3). There were slight variations in the total number of screenings per week, with the fewest screenings being taken over the July 4 holiday week. The number of high-risk screenings increased in the final week as a result of a confirmed positive case within the study population.

**Figure 9:**
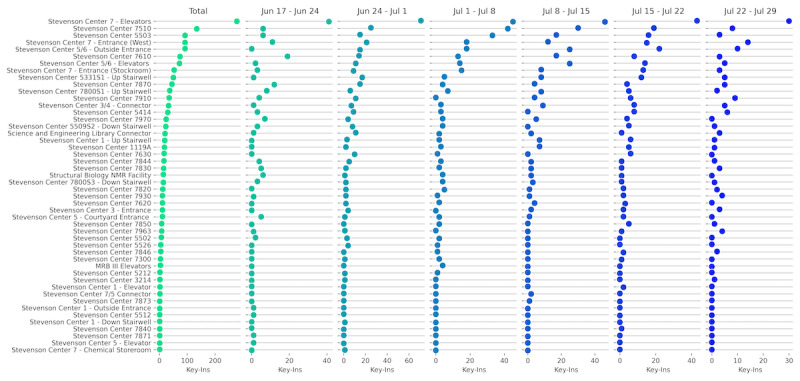
Key-ins per location for each week.

Key-ins were performed by 32 different users and occurred at 48 unique locations. Accounting for the variation in dates of user account creation, on average, users keyed in 6.75 times per week (Multimedia Appendix 4). Only 67% (n=48) of the 71 locations with flyers were actually used by the participants. The 5 most commonly visited locations accounted for almost 50% (688/1410) of all key-ins (Figure 9). Several of the most frequented locations are expected: the most central elevator at the heart of Stevenson Center Building 7 (the home building for the majority of our users) and multiple building entrances. While several locations saw a substantial increase or decrease in usage from week to week, possibly in part due to our enrollment size being small and our results therefore subject to the fluctuations of individual schedules, the rate of usage at the most frequented locations remained roughly constant from week to week.

**Figure 10:**
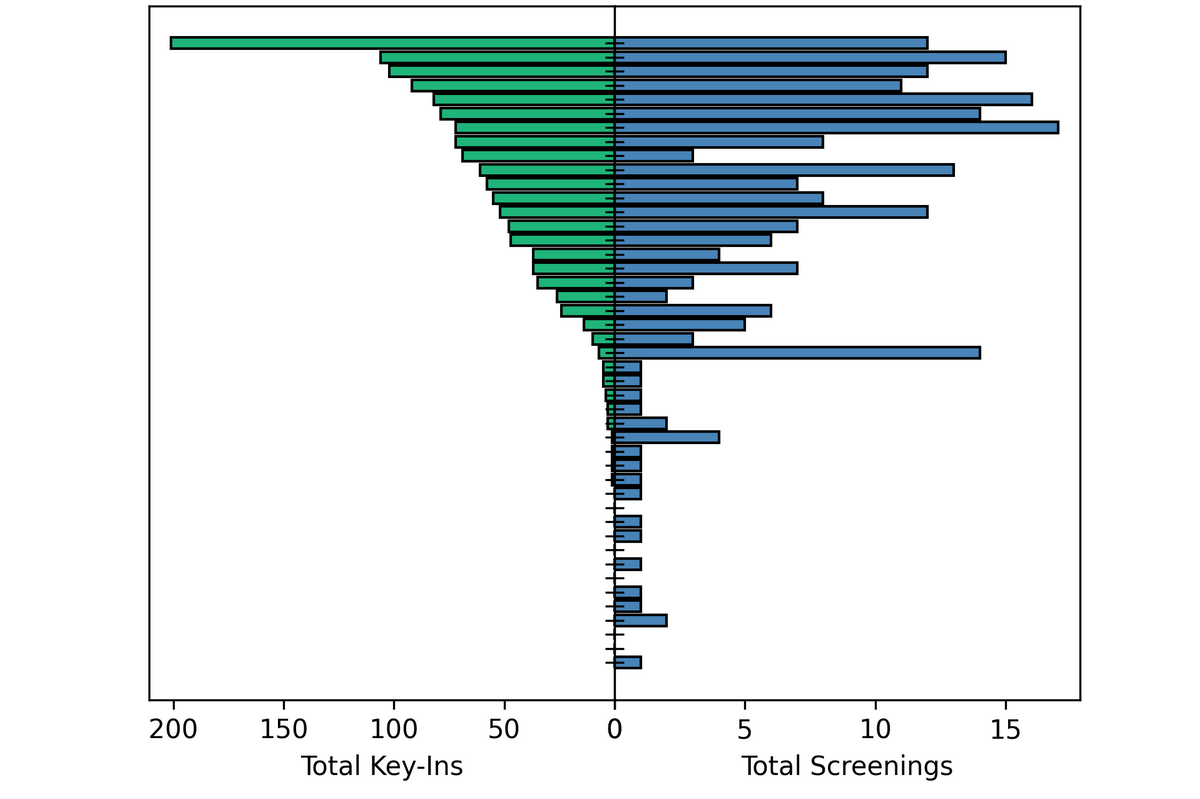
A comparison of the total key-ins and screenings for each user in the pilot study. The total key-ins per user are shown on the left (green), while the number of screenings is displayed on the right-hand side (blue).

While Figure 7 suggests a proportional relationship between the usage of the self-assessment tool and the key-in feature, app usage was not evenly distributed among our users, as would be expected with a new technology [42]. Figure 10 shows the total key-ins and screenings for our users (each user being a horizontal line on the y-axis), sorted by the number of key-ins for that user. The top of the graph shows that we had several high-volume participants who utilized both features of the app frequently. Conversely, there were 5 accounts that never keyed in or took a self-assessment (2 of which never logged in after creating an account). A total of 10 users did not use the app beyond their first self-assessment. Interestingly, several users appear to have used the self-assessment tool disproportionately compared to their use of the key-in feature. This is possibly tied to the increase in remote work for those individuals relative to their on-campus hours, or potential concerns over privacy after initial usage of the app.

### Contact Tracing

The potential for interactions, even in a small number of people, is large (Multimedia Appendix 5). Our app has two approaches for contact tracing using this individualized spatiotemporal data: manual and automatic. In manual contact tracing, administrators can search for a user by name or email address, find the locations that these users have visited, and identify any other users that keyed in to these locations within the overlap window (Multimedia Appendix 6). In automatic mode, a contact tracing case is created after each self-assessment that indicates either symptoms of or potential exposure to COVID-19. Every case consists of a POI (the user that took the self-assessment), locations of interest (locations that the POI keyed in to during their case window), and contacts of interest (other users that keyed in to locations of interest within a predefined “overlap” window). While the manual mode is designed to augment traditional contact tracing with digital data, automatic contact tracing can be used to streamline this process by compiling lists of contacts and locations, and potentially automating some tasks (notifications, cleaning schedules, etc).

Over the duration of the study, 25 self-assessments indicated either symptoms of or potential exposure to COVID-19. The 25 cases came from 8 unique users, and in 19 of the cases, the POI keyed in to a location on campus after their assessment indicated they were NOT CLEAR. In the event of an at-risk self-assessment, our app makes a prominent recommendation that users isolate and assists them to identify testing locations nearby (Figures 4B and 6D), but our pilot did not have the authority to keep users away from campus. For the purposes of this pilot study, we did not collect self-reported information from users on whether they were tested after receiving a NOT CLEAR status.

**Figure 11:**
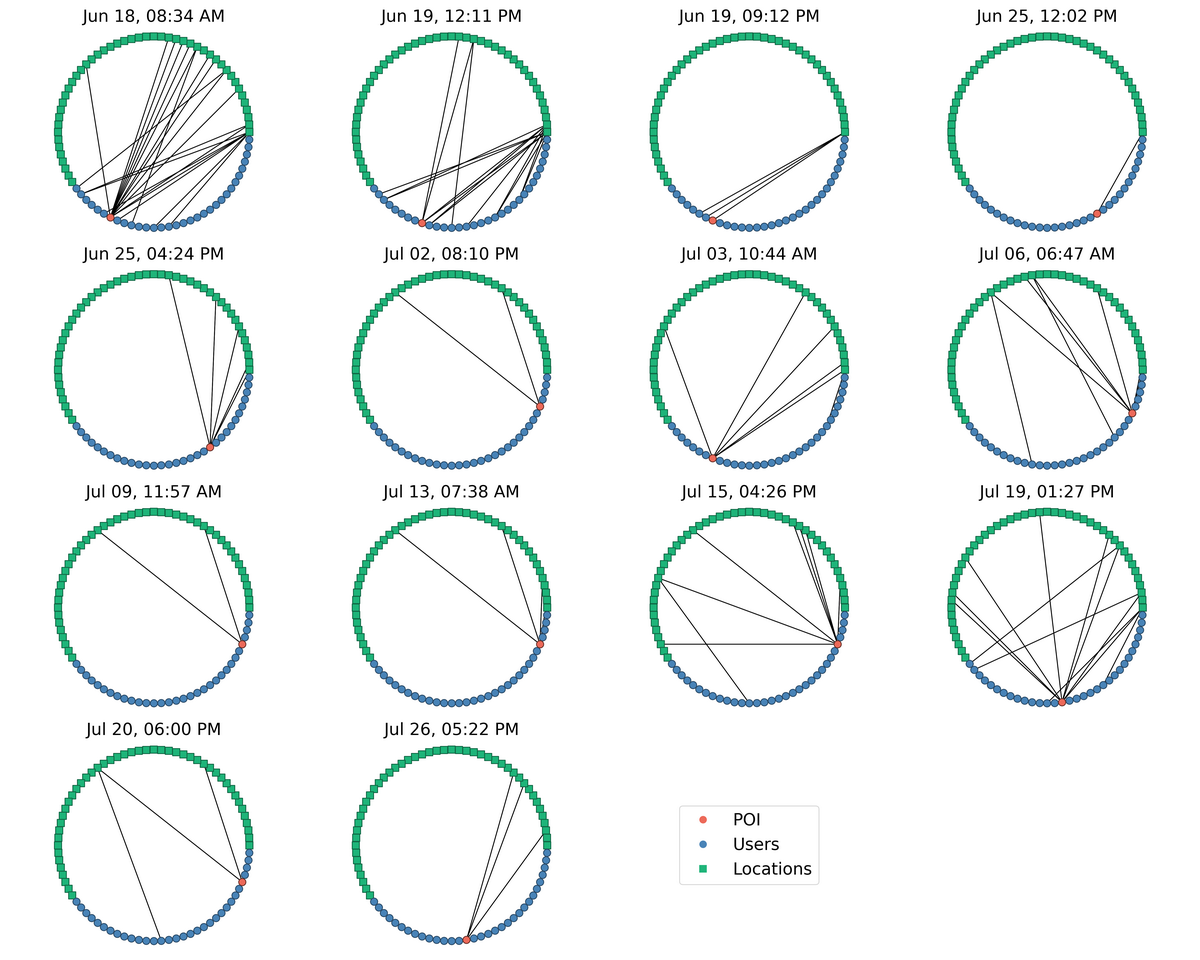
A network connectivity diagram showing person-of-interest (POI) key-ins to locations of interest, as well as key-ins by other users at those same locations within the overlap window.

Of the 19 cases where the POI keyed in at least once on campus, there were 26 unique locations affected. The cases are summarized in a network chart (Figure 11) where each green square represents a location, blue circles represent users, and the red circle represents the POI. Lines connecting the POI and locations represent key-ins at those locations during the case window. Lines connecting other users and these locations represent key-ins during the overlap window. For brevity, we have not included any cases where POIs had multiple NOT CLEAR self-assessments within the same case window in Figure 11. Several cases had no overlapping users, while in others the density of connected locations at risk and contacts at risk was markedly increased.

All digital contact tracing algorithms have parameters that must be explored in order to optimize accuracy. In our automated algorithm, the following parameters could be adjusted: the reverse case window period, the forward case window period, and the overlap window. We explored the sensitivity of our results to each of these parameters. While the total number of cases is fixed by the results of the users’ self-assessments, as expected, the key-ins per POI, number of locations at risk, and number of contacts at risk all increase as these windows increase (Multimedia Appendices 7-9).

## Discussion

### Principal Findings

The COVID-19 pandemic has brought disease control strategies to the general public’s attention. The need for robust contact tracing is broadly understood, particularly as states, and consequently, educational institutions, move through their phased reopening plans. While the need is agreed upon, reports of the lack of contact tracing infrastructure highlight the space where digital contact tracing tools can be useful. In this work, we describe a pilot study of MyCOVIDKey, a digital contact tracing app. The app consists of recurring self-assessments and user key-ins, whereby a user scans a unique barcode to indicate their presence at a location. A 6-week pilot study took place within the Stevenson Center Science and Engineering Complex, on the Vanderbilt University campus in Nashville, TN. The pilot study was successful, and after app revisions based on user feedback (presented in detail in Scherr et al [39]), MyCOVIDKey will be ready for wider-scale deployment to campus and office settings. In this study, we found two clear purposes that could be addressed with digital interventions like MyCOVIDKey: (1) the identification of contacts of a POI who could have potentially been exposed and (2) the identification of locations that POIs visited that may be candidates for enhanced cleaning. Both are expected to remain key needs throughout the duration of the pandemic, even after the distribution of a vaccine.

While the postpilot survey results are analyzed separately in greater detail [39], it is worth noting that from this data it was clear that the majority of MyCOVIDKey users were young: 73.3% (22/30) of respondents were aged 20-30 years, while 20% (6/30) were aged 30-40 years and 6.66% (2/30) were 41 years of age or older. In addition, 77% (23/30) of our users were graduate students engaged in research. This cohort represents a biased group that is more likely to adopt newer technologies, confident in utilizing mobile phone apps, and interested in participating in the pandemic response. As such, our users may have different usage patterns, concerns, and preferences than a larger campus population, or even more so compared to a nonacademic audience. This selection bias was unavoidable considering the location and timing of the study, and its impact should be further studied on larger populations.

As we developed our app, we made several key decisions that should be further explored. Some implementations of COVID-19 self-assessments for return-to-work purposes do not allow users to access buildings or floors of their office space if they exhibit symptoms. This study took the alternative approach of allowing users to continue keying in with an at-risk self-assessment. This decision was made primarily for two reasons: (1) our pilot study did not have the authority to deny the participants entry into buildings or send them home from work, as those decisions were left to the reopening guidelines from the university; (2) we believed that there was the likelihood that users with at-risk self-assessments would continue to enter the building, regardless of their MyCOVIDKey status, and it was preferential to obtain data on their locations while at risk. This decision, albeit with a small sample size, was validated by the result that 19 of the 25 NOT CLEAR statuses still keyed in on campus, which indicates minimal behavioral change occurred, in this study, based on the user’s status. Ideally, symptomatic individuals would follow the app’s recommendations and isolate until they have either received a negative diagnostic test result or their window for transmission has lapsed. While this could have resulted from the perception of a lack of enforcement authority of the study, it could have also been explained by any diagnostic testing results that users may have obtained during the study. We are unable to draw conclusions on compliance since we did not actively seek input on diagnostic testing results after a NOT CLEAR status. This lack of diagnostic backing for self-reported symptoms may have introduced some amount of information bias due to the reliance on user memory and self-reporting. Regardless, this highlights a clear distinction between contact tracing software and a “passport” that allows entry if you meet checkpoint criteria. Given the level of asymptomatic spread of COVID-19, we believe that such passports are meaningful when tied to recent diagnostic testing—and considerably less useful with self-assessments alone. This distinction becomes even more critical when entrance to a location is tied to an incentive, for instance financial incentives at work or social or educational incentives on campuses.

In this study, we noted several parameters in our automatic contact tracing algorithm that must be tuned. Using the CDC’s guidelines of 6 feet or less for 15 minutes or more to denote a “true” contact event, there will always be false positives and false negatives associated with digital contact tracing tools. False positives generated by digital contact tracing tools will increase the workload for manual contact tracers. For instance, increasing the overlap window or the case window parameters of our system will increase the number of locations and potential contacts that need to be traced. This could potentially become overwhelming for manual contact tracers in large organizations or in populations where there is a relatively high positivity rate. In contrast, false negatives from digital contact tracing tools will rely on manual efforts to correctly be identified, or risk not knowing forward disease transmission. We therefore recommend that the sensitivity and specificity with our system, and likely other digital contact tracing tools, be optimized depending on the population size, the local disease prevalence, and the level of automation allowed by contact tracing. One option that could be implemented in parallel to relieve burden on manual contact tracing efforts is to allow automated digital tools to only take action based on events that can be classified with a high degree of confidence. Based on the necessary tuning of parameters, it is our belief that digital contact tracing tools still serve best as a complement to manual contact tracing efforts, and not as a standalone replacement. This is not to minimize their importance. In fact, we believe they are an essential supplement to the realistic infrastructure constraints observed with manual contact tracing. When used appropriately, they can reduce the burden facing manual contact tracers by offloading certain inquiries and tasks.

While all contact tracing tools share the same goals, our technology has some notable differences from other approaches. MyCOVIDKey does not rely on Bluetooth or GPS to identify potential contact events; rather it relies on users to scan a barcode that identifies locations that they enter. This has technical advantages over the latter technologies, namely its ability to distinguish users in the same room from those separated by walls or even on different floors, as well as enhanced user privacy. Its primary disadvantages are that it does not capture potential exposures that could occur in transit between locations, and that it requires users to actively participate rather than rely on a continuous, automated data stream. While passive data collection is attractive to users due to the minimal effort required, it does come with increased privacy concerns—particularly as the sale of user location data for marketing purposes has become commonplace [43-47].

An additional limitation of our platform compared to others is the inability to determine how long users stayed at a particular location or to determine their proximity to other users. Since users are only asked to key in upon entrance, and not exit, in the current version of the software, determining the overlap window’s forward time limit is a challenge. Using the default overlap window of 30 minutes, our results for contacts of interest would count relatively harmless events like the keying in of 2 users to an elevator 25 minutes apart. However, it would miss events that may be noteworthy; for instance, key-ins to a classroom or laboratory that take place an hour apart, but where the POI has not yet left the room. A simple improvement is to allow organizations to define specific windows of interaction for different types of locations. This could more accurately reflect, for instance, that the timescales spent in elevators (seconds) is fundamentally different than time spent in classrooms (minutes) and in research labs (hours). An alternative approach to remedying this would be to ask users to key in at stations upon exiting as well. While this would place more burden on users and may therefore negatively affect continued usage outside of the consistent user group, it would provide the needed closure on user activity to ensure a more prescriptive assessment of risky interactions.

In this study, we did not ask users with a NOT CLEAR status if they received diagnostic testing to confirm or override their status in the app. The primary objectives of this study were to evaluate the usage of the platform and not to compare self-reported symptoms with diagnostic testing. Therefore, users who were identified as NOT CLEAR and considered a POI may have received a negative result from a SARS-CoV-2 diagnostic test and would be allowed to safely return to campus. While inclusion of this information has obvious utility, as in the aforementioned case, its implementation may be (depending on the disclosing party, any verification of the test results with the provider, and the user’s parent organization that is utilizing this information) subject to regulation by the Health Insurance Portability and Accountability Act. In our postpilot survey, we did ask users about their experiences with COVID-19 testing. While explained in greater detail in our analysis of the postpilot survey data [39], one MyCOVIDKey user did report a positive diagnostic test for SARS-CoV-2. Importantly, MyCOVIDKey was able to accurately identify this person as a POI (this user was symptomatic, and their self-assessment indicated high risk), the locations they had visited in the buildings, and their contacts of interest. Given that the on-campus population was small due to local government safer-at-home orders and the university’s emphasis on remote work where possible, the university’s manual contact tracing team had sufficient capacity to handle this case. This reiterates that while the study setting was ideal for this pilot trial, MyCOVIDKey is perhaps most appropriate for settings where contact tracing infrastructure is not able to handle the volume of cases without additional support.

The usage of MyCOVIDKey during the pilot period closely followed the diffusion of innovation theory. The pilot had a group of early adopters that eagerly took on the platform. This core group was responsible for driving early usage and likely had a positive impact on encouraging new sign-ups and continued usage among their peers. Our pilot study launched without an organizational mandate or directive to use our app. In the absence of this, we made use of a weekly raffle to incentivize usage and participation. Businesses and higher-education institutes have the authority to give employees and students such an order. Forced mandates, however, could be met with resentment and resistance that would negatively affect their usage and undermine their objectives. So while it is understood that there is a critical threshold of users that must be reached in order for these tools to be effective [18], organizations must carefully balance the concerns of their members with public health needs when deciding how to meet this threshold.

### Conclusion

Contact tracing is an essential component of any response to an epidemic, and digital contact tracing platforms are poised to play a large role in the current COVID-19 pandemic. In this paper, we have described one such tool, MyCOVIDKey, and a pilot evaluation of its usefulness in a university setting. We were able to identify several potential roles of digital contact tracing supplements, including the identification of potential contacts of at-risk individuals and resource allocation for local testing and building facilities management. While our platform, and these results, are directly applicable to campus communities, they are extensible to the reopening of businesses and communities at large as well. Although more studies are necessary to understand how variations in both the district and national levels could affect uptake in disparate populations and to develop effective mobile health implementation approaches [48], digital health interventions will likely be utilized worldwide. All organizations must make decisions on how best to integrate these tools into existing pandemic response infrastructure, as well as how to address potential concerns over data ownership and stewardship, while still reaching a critical threshold of necessary users for these tools to be effective. With a better understanding of the broader utility of MyCOVIDKey and apps like it, refinements will be made to simultaneously enhance the app’s usability and security. Our pilot study shows that MyCOVIDKey can address the needs of many academic institutions and businesses as they begin to reopen.

## Appendix

Multimedia Appendix 1 The user privilege hierarchy for MyCOVIDKey, along with the data that each user class can access.

Multimedia Appendix 2 The home screen of MyCOVIDKey displays information about the user’s current MyCOVIDKey status, allows users to perform self-assessments, key in to new locations, and view simple usage statistics. Certain features are disabled, and the text is adjusted to reflect a user’s current status: (A) NO STATUS for new accounts, and (B) EXPIRED status.

Multimedia Appendix 3 An overview schematic of the MyCOVIDKey automated contact tracing algorithm.

Multimedia Appendix 4 The probability density of key-ins and screenings per week.

Multimedia Appendix 5 A network graph of all key-ins, regardless of time. Green squares represent locations, blue circles represent individual users, and lines represent key-ins from a user at a specific location.

Multimedia Appendix 6 The manual contact tracing portal provides contact information for the POI, the locations that the user keyed in to during the search window, as well as overlapping users at those locations.

Multimedia Appendix 7 A sensitivity analysis of how varying the reverse (left) and forward (right) case windows affects the amount of POIs that keyed-in during their case window.

Multimedia Appendix 8 A sensitivity analysis of how varying the reverse (left) and forward (right) case windows affects how many key-ins had overlapping users.

Multimedia Appendix 9 A sensitivity analysis of how varying the contact overlap window affects the number of overlapping users (contacts of interest).

## Abbreviations

CDC: Centers for Disease Prevention and Control
POI: person of interest
QR: quick response code
