# Supplementary figures and images for: Understanding On-Campus Interactions With a Semiautomated, Barcode-Based Platform to Augment COVID-19 Contact Tracing: App Development and Usage
Source: JMIR Mhealth Uhealth. 2021 Mar 26;9(3):e24275. doi: 10.2196/24275 (PMC8006900; doi:10.2196/24275)

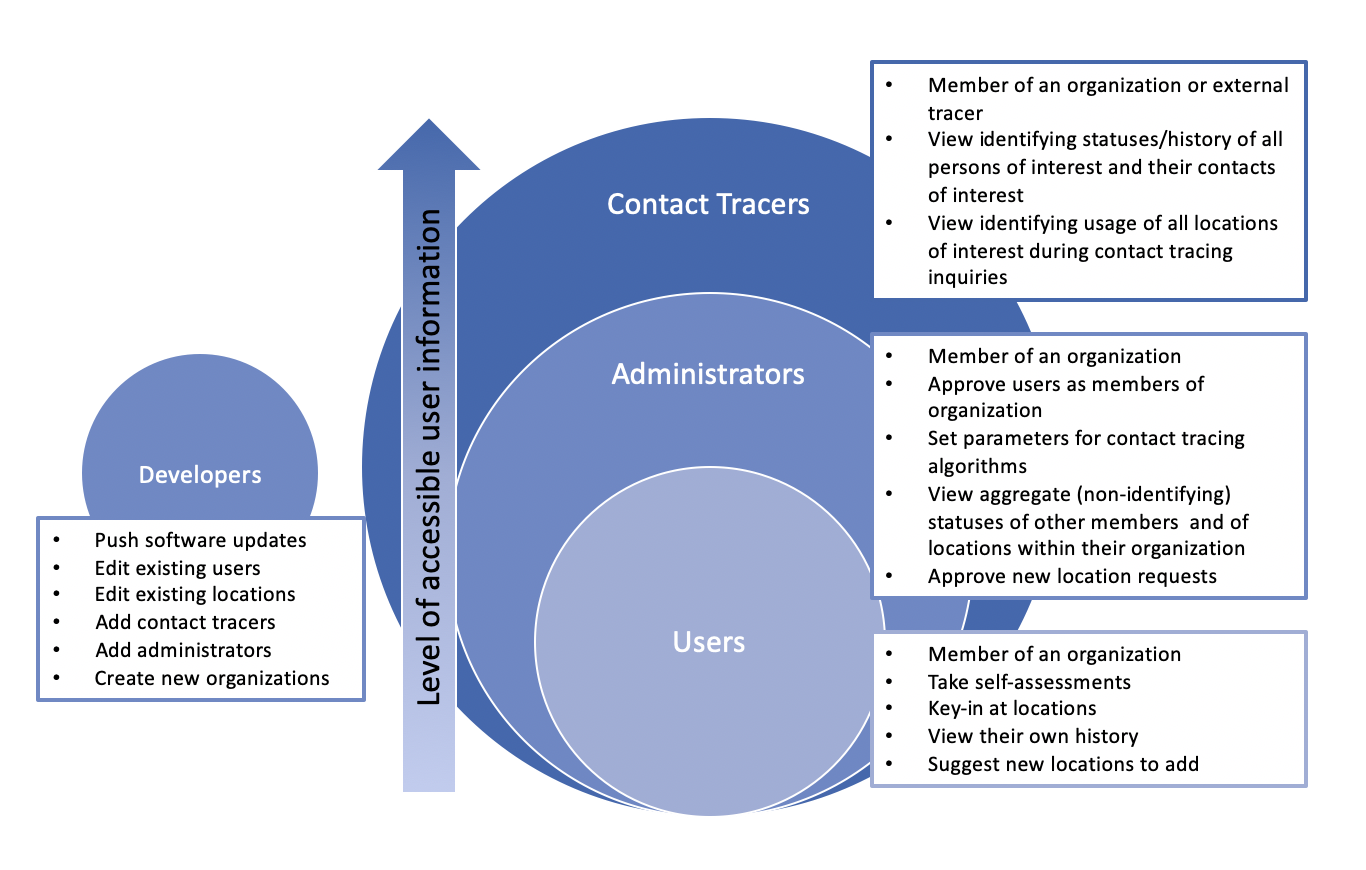

Supplement: Multimedia Appendix 1 [file mhealth_v9i3e24275_app1.png]

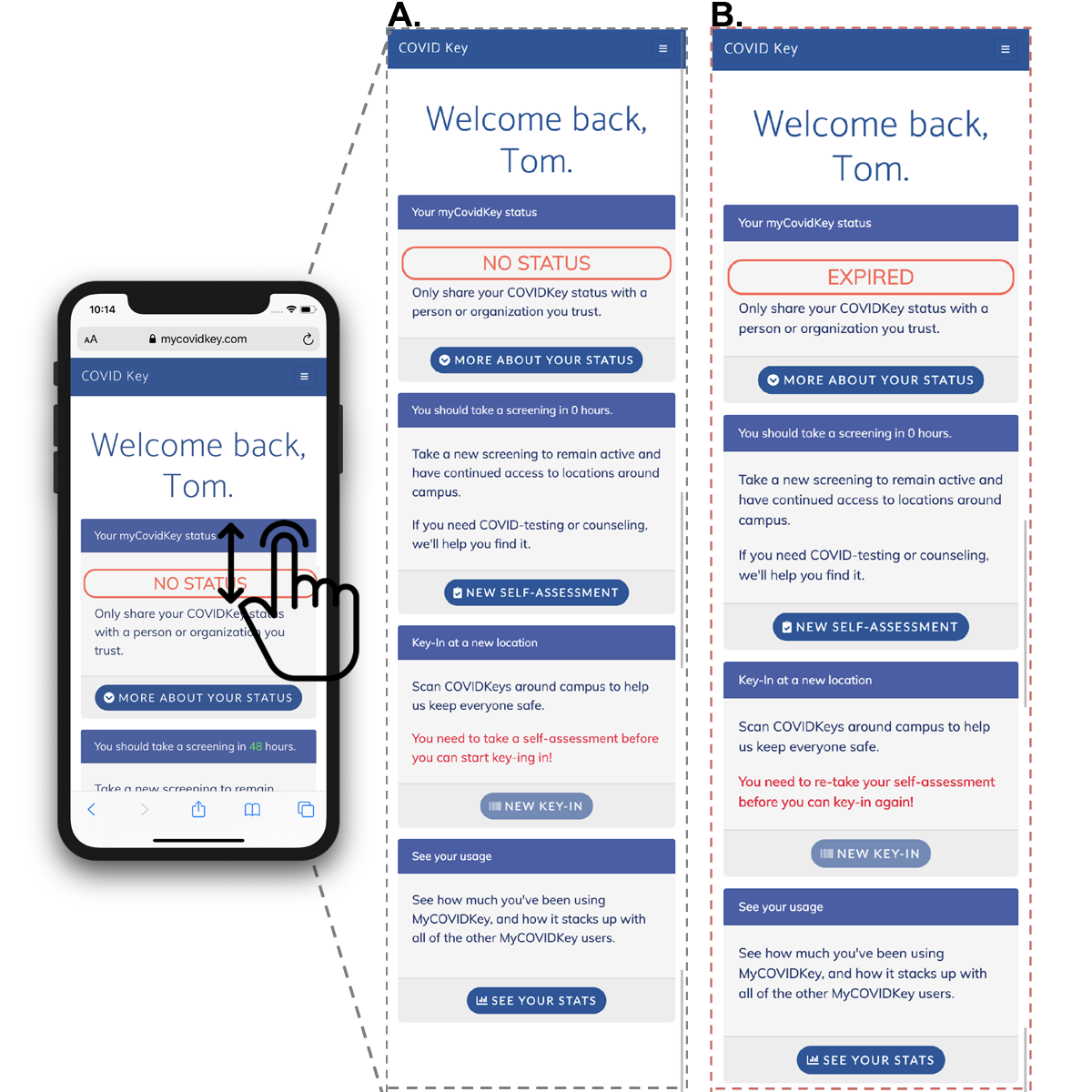

Supplement: Multimedia Appendix 2 [file mhealth_v9i3e24275_app2.png]

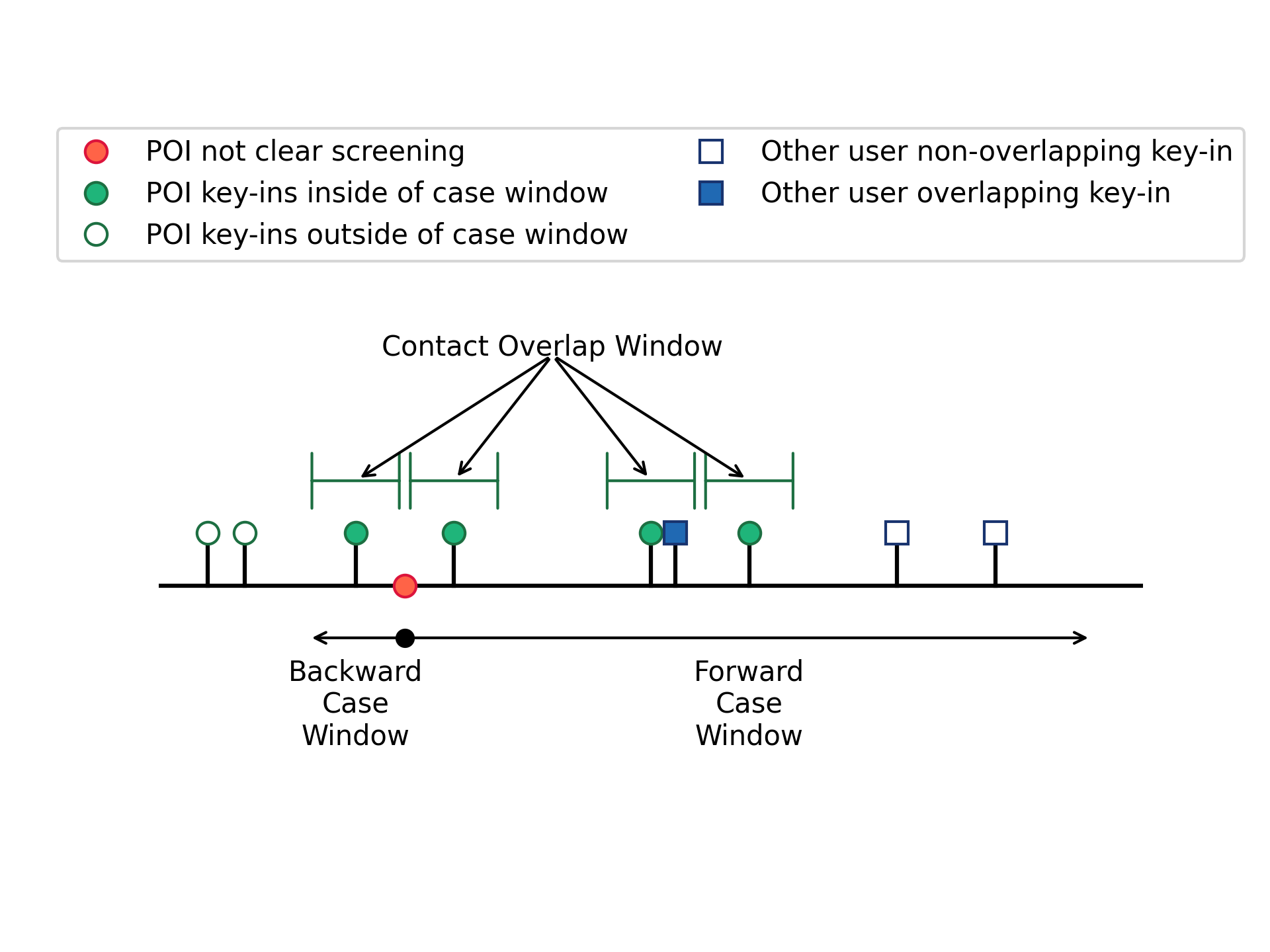

Supplement: Multimedia Appendix 3 [file mhealth_v9i3e24275_app3.png]

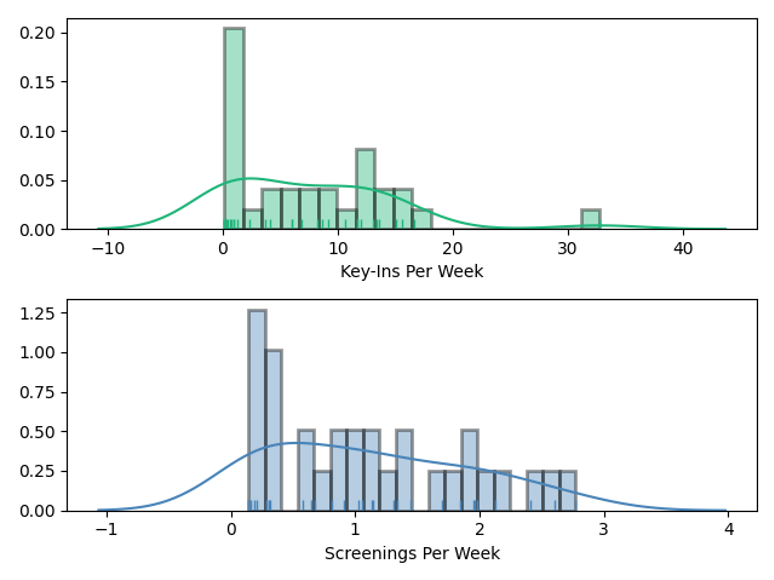

Supplement: Multimedia Appendix 4 [file mhealth_v9i3e24275_app4.png]

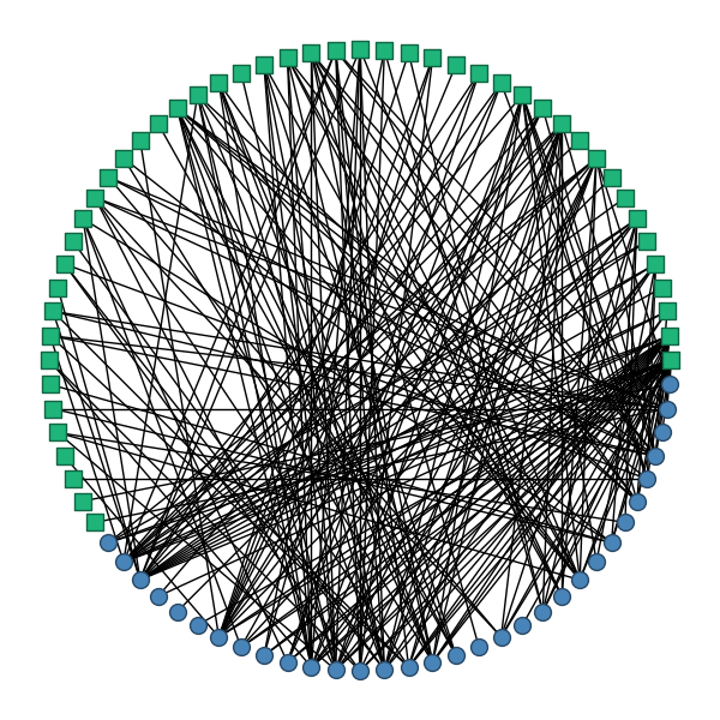

Supplement: Multimedia Appendix 5 [file mhealth_v9i3e24275_app5.png]

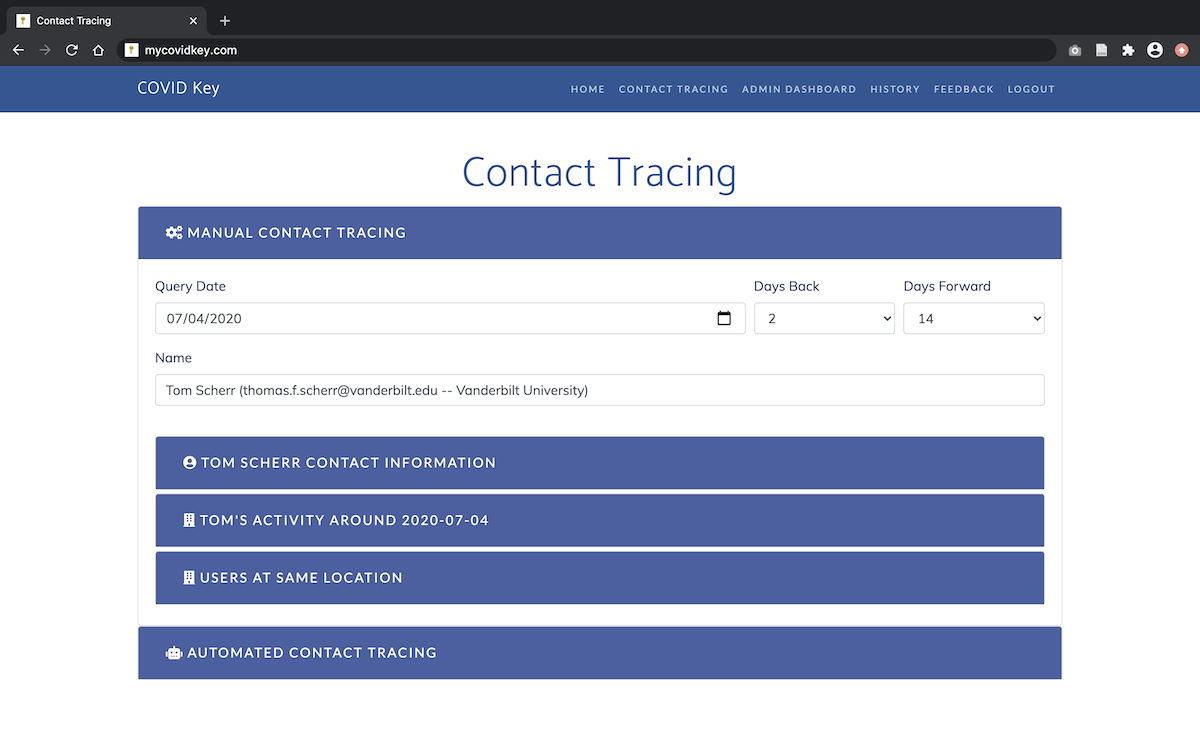

Supplement: Multimedia Appendix 6 [file mhealth_v9i3e24275_app6.png]

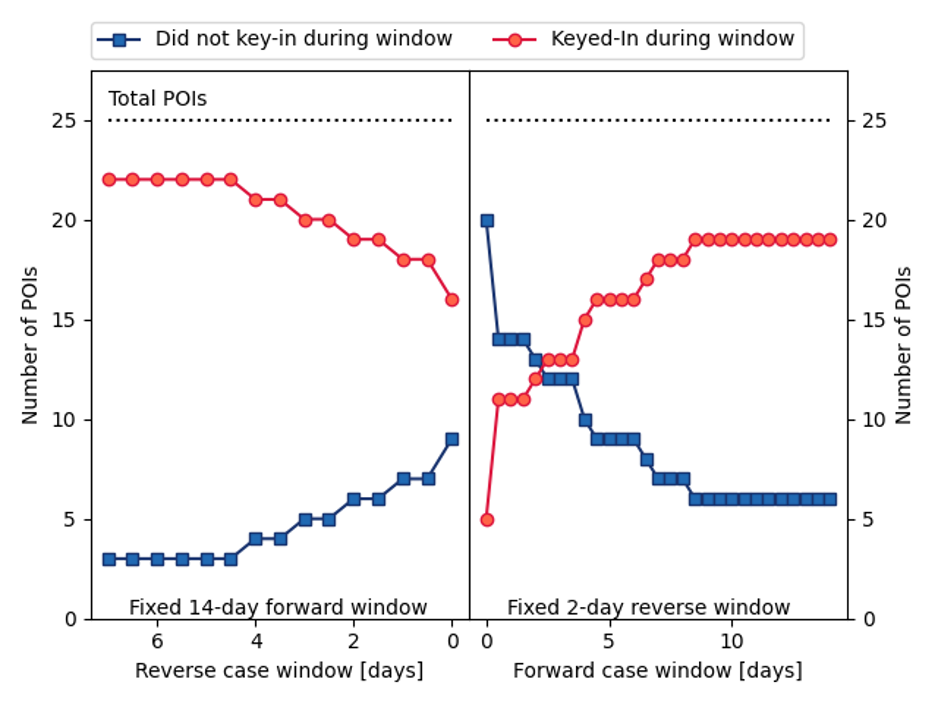

Supplement: Multimedia Appendix 7 [file mhealth_v9i3e24275_app7.png]

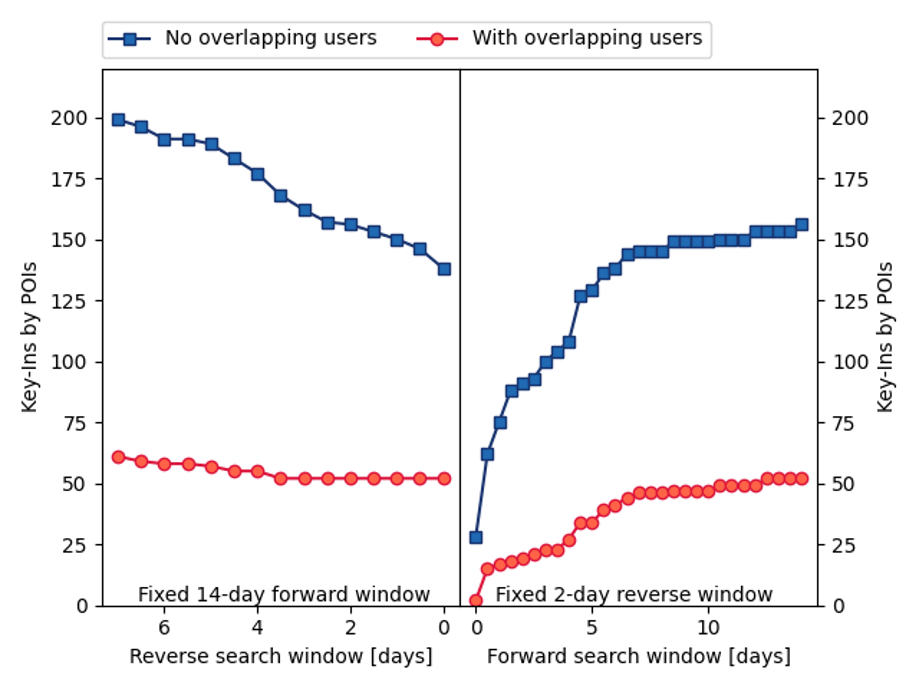

Supplement: Multimedia Appendix 8 [file mhealth_v9i3e24275_app8.png]

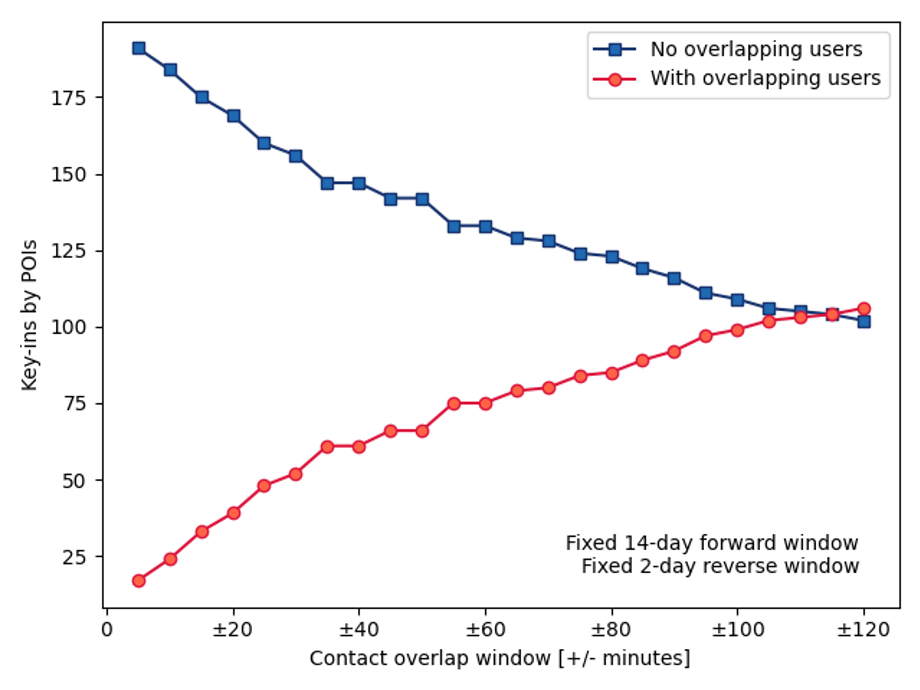

Supplement: Multimedia Appendix 9 [file mhealth_v9i3e24275_app9.png]
